# Hybrid origin of European Vipers (*Vipera magnifica* and *Vipera orlovi*) from the Caucasus determined using genomic scale DNA markers

**DOI:** 10.1186/s12862-016-0647-7

**Published:** 2016-04-12

**Authors:** Oleksandr Zinenko, Michael Sovic, Ulrich Joger, H. Lisle Gibbs

**Affiliations:** The Museum of Nature at V.N. Karazin Kharkiv National University, Trinkler str. 8, Kharkiv, 61058 Ukraine; Department of Evolution, Ecology, and Organismal Biology, The Ohio State University, Aronoff Laboratory, 318 W. 12th Avenue, Columbus, OH 43210 USA; The Ohio Biodiversity Conservation Partnership, The Ohio State University, Aronoff Laboratory, 318 W. 12th Avenue, Columbus, OH 43210 USA; Staatliches Naturhistorisches Museum Braunschweig, Gausstrasse 22, Braunschweig , D-38106, , Germany

**Keywords:** RADSeq, Patterson D-statistic, Bayesian clustering, Gene introgression, *V. kaznakovi*, *V. renardi*

## Abstract

**Background:**

Studying patterns of introgression can illuminate the role of hybridization in speciation, and help guide decisions relevant to the conservation of rare taxa. *Vipera magnifica* and *Vipera orlovi* are small vipers that have high conservation status due to their rarity and restricted distributions in an area of the Caucasus region where two other related species are present – *V. kaznakovi* and *V. renardi.* Despite numerous observations of hybridization between different species of small vipers, and the potential of a hybrid origin for *V. magnifica* and *V. orlovi* based on their distribution with respect to *V. kaznakovi* and *V. renardi*, hypotheses of a hybrid origin have not been formally tested. Here we generate genomic-scale data by performing next generation sequencing of double digest restriction-site associated DNA libraries, and use these multilocus data to test whether these two species are of hybrid origin.

**Results:**

We generated over nine hundred loci for 38 specimens of six taxa, and analysed the dataset using Bayesian clustering and multivariate methods, as well as Patterson D-statistics, which can distinguish between incomplete lineage sorting and introgression as explanations for shared polymorphism. The results demonstrate a pattern of historical admixture in the two purported hybrids that is consistent with past gene flow from *V. renardi* into *V. kaznakovi*. The average admixture proportion in individuals was low (6.39 %) in the case of *V. magnifica*, but was higher in *V. orlovi* (19.02 %). We also show that the specific individual samples used in D-statistic tests can have a significant impact on inferences regarding the magnitude of introgression, suggesting the importance of including multiple individuals in these analyses.

**Conclusions:**

Our results support the conclusion that both *V. orlovi* and *V. magnifica* had formed through a hybridization event between *V. kaznakovi* and *V. renardi*. Given a low proportion of admixture and absence of clear ecological and morphological differences *V. magnifica* should be treated as a marginal population of *V. kaznakovi*. Further studies that include analyses of ecological segregation of *V. orlovi* from parental taxa and search for evolutionary consequences of hybridisation would clarify if *V. orlovi* is a distinct hybrid species. Until this we recommend preserving the current taxonomy and protection status of *V. orlovi*.

**Electronic supplementary material:**

The online version of this article (doi:10.1186/s12862-016-0647-7) contains supplementary material, which is available to authorized users.

## Background

Hybridization is increasingly recognized as an important evolutionary process that can have diverse outcomes with respect to biodiversity. For example, gene introgression can act to increase diversity by rapidly generating novel genetic variation upon which selection can act, potentially leading to speciation [1, 2]. In contrast, hybridization caused by human habitat disturbance may reduce biodiversity through the genetic fusion of species (reverse speciation) or the genetic dilution of a rare taxon by a more abundant one [3, 4].

In snakes, there are increased observations of hybridization between species in phylogenetically-distinct groups [4–11]. Hybridization has been widely documented for European vipers from the genus *Vipera* in both nature and captivity, and may be especially common in the subgenus *Pelias*, a monophyletic group that consists of approximately 20 species of small venomous snakes found in the temperate zone of Eurasia [12]. Hybrids within *Pelias* have been reported in a number of studies including those based on karyotype, osteological, and molecular methods [13–22, Zinenko, unpublished]. Ancient introgression has also been proposed as a factor influencing the evolution of some viper species (i.e. *Vipera kaznakovi* Nikolsky, 1909, *V. darevskii* Vedmederja, Orlov and Tuniyev, 1986, *V. dinniki* Nikolsky, 1913 and *V. eriwanensis* (Reuss 1933)), but as yet no direct evidence beyond similarities in external morphology has been provided [23].

*V. renardi* (Christoph 1861) and *V. kaznakovi* are two widely accepted complexes of viper species that have distributions that meet along the northern slope of the Caucasus [23–29]. Snakes of the *V. kaznakovi* complex are endemic to the Caucasian region and are found in two different habitat types: forests of the northwestern and southern slopes of the Caucasus and high altitude open meadows of alpine zones. Snakes of the *V. renardi* complex consist of several taxa living in lowland or dry mountain grasslands which have expanded their range in Northern Caucasus through natural or artificial corridors resulting in a zone of contact between these two groups.

*V. orlovi* Tuniyev et Ostrovskikh, 2001 and *V. magnifica* Tuniyev et Ostrovskikh, 2001 were recently described on the basis of morphological differences and are considered part of the *V. kaznakovi* group [30]. Both species have features such as intermediate geographic location, habitat, and morphology that suggest they could be hybrid lineages that originated due to historical and/or ongoing hybridization between *V. kaznakovi* and *V. renardi*. In addition, mitochondrial DNA haplotypes from *V. magnifica* and *V. orlovi* are shared with *V. kaznakovi* [29], further pointing toward hybridization.

Understanding the evolutionary origin of these species would help to understand the role that interspecific gene flow may play in the process of radiation and local adaptation in vipers. In addition, since both species are included on the IUCN list of threatened species and are locally protected [31–33], clarification of their evolutionary and taxonomic status is needed to accurately identify targets for conservation efforts.

Genetic markers traditionally used to assess hybridization between species (e.g. AFLP, microsatellites, individual nuclear genes) assay variation at few positions in the genome, can show limited levels of informative variation, and may require *de novo* development for use in non-model species [34–37]. The recent availability of next generation sequencing technologies has opened a door for rapidly genotyping many individuals at thousands of potentially informative loci throughout the genomes of non-model organisms [38]. This has led to the availability of more comprehensive datasets for studies of hybridization and genetic introgression. Restriction site associated DNA sequencing (RADseq) is one specific method that takes advantage of these sequencing technologies to generate data from a large number of unlinked independent loci dispersed across genome, while reducing the need for a reference genome to identify orthologs [38]. This type of genetic information is increasingly being applied for population genetic and evolutionary analyses in a wide range of organisms [39–44].

Along with advances in methodologies for generating data have come novel analytical techniques that take advantage of genomic-scale datasets. These have great potential for studies of introgression because they provide the ability to distinguish between introgression and ancestral polymorphism, which is a significant issue when evaluating potential hybridization between closely related taxa. One example is Patterson’s D-statistic, which was first developed and applied to detect hybridization between different Hominid lineages based on genomic data [45, 46]. This approach has recently been used with RADSeq data to detect gene flow and identify introgressing loci in several non-model systems [41, 43, 44, 47].

In this paper, we use the modification of RADSeq data – ddRAD (double digest restriction-site associated DNA) to investigate the evolutionary origin of the purported hybrid taxa *V. magnifica* and *V. orlovi*. We assess these questions by 1) evaluating patterns of genetic structure among these groups using genetic clustering methods, and 2) testing for patterns of genetic introgression among the groups using ABBA/BABA tests and associated Patterson D-statistics. Our results support the hypothesis that these two groups arose through hybridization between the steppe viper *V. renardi* and the Caucasian viper *V. kaznakovi*. We discuss the results as they relate to the evolutionary history of these snakes and also in the context of their relevance to the conservation of these species.

## Results

### Identification of loci

Approximately 3.3 x 10^6^ reads were obtained for the 38 individuals sequenced. Analyses with and without the Outgroup samples resulted in the identification of 3651 and 1959 polymorphic loci, respectively. Of the 3651 polymorphic loci obtained when outgroup samples were included, 977 were genotyped in all of the samples, and were used for analyses.

### Genetic variation

The four taxa of vipers analysed in this study show slightly different patterns of genetic variation at RAD loci (Table 1). *V. renardi* is characterized by the lowest allelic richness but the highest number of private alleles. At the same time, it is the only group with observed overall heterozygosity that is lower than expected. Both *V. magnifica* and *V. orlovi* have relatively low numbers of private alleles, but observed heterozygosity that is higher than expected when compared to *V. kaznakovi* and *V. renardi*. Finally both observed heterozygosity and allelic richness are highest in *V. orlovi*.

**Table 1:** Average number of alleles K, allelic richness AR, number of private alleles (Priv) and observed and expected heterozygosity (Ho and He) in samples of four species of Vipers

	N	K	AR	Priv	Ho	He
*V. kaznakovi*	8	1.58 ± 0.65	1.32 ± 0.39	112	0.17 ± 0.22	0.17 ± 0.20
*V. magnifica*	2	1.29 ± 0.48	1.29 ± 0.48	14	0.18 ± 0.32	0.12 ± 0.20
*V. orlovi*	12	1.77 ± 0.67	1.42 ± 0.41	69	0.25 ± 0.25	0.22 ± 0.20
*V. renardi*	12	1.61 ± 0.66	1.22 ± 0.29	399	0.10 ± 0.15	0.12 ± 0.17

Values are given as means ± SD

Fst values were significantly greater then zero for all pair-wise comparisons of taxa (Table 2). Pairs which included *V. kaznakovi*, *V. magnifica* and *V. orlovi* are equally distinct from each other with Fst values close to 0.2, whereas *V. renardi* is much more distinct from all three of the other taxa (pairwise Fst values for comparisons including *V. renardi* were between 0.64 and 0.75).

**Table 2:** Pairwise Fst values calculated in Arlequin [64] for two parental species, *V. kaznakovi* and *V. renardi*, and two purported hybrid groups, *V. magnifica* and *V. orlovi*

	*V. kaznakovi*	*V. magnifica*	*V. orlovi*	*V. renardi*
*V. kaznakovi*	----------			
*V. magnifica*	0.18	----------		
*V. orlovi*	0.17	0.20	----------	
*V. renardi*	0.72	0.75	0.64	----------

All values were significantly greater than zero based on a permutation test (*p* < 0.05)

**Fig. 1:**
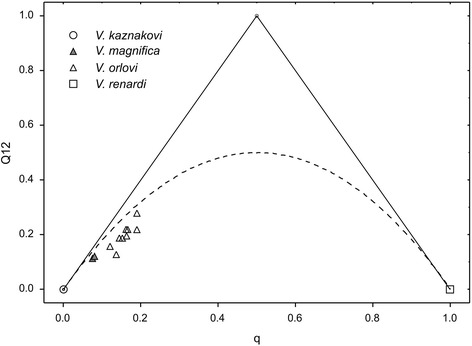
Scatterplot showing the relationship between global genetic ancestry q estimated using 133 biallelic loci fixed for different alleles in parental species *V. kaznakovi* and *V. renardi* and the inter-source population ancestry for these loci Q12 [48]. Symbols correspond to individuals. Lines indicate the maximum level of inter-source population ancestry given global genetic ancestry q (solid lines) and the expected levels of Q12 if the hybrid population is in Hardy-Weinberg equilibrium (dashed line). These results provide no evidence for ongoing gene flow from the parental groups *V. kaznakovi* and *V. renardi* to the proposed hybrid groups *V. magnifica* and *V. orlovi*

A restricted dataset of 133 biallelic loci fixed between samples of *V. kaznakovi* and *V. renardi* showed that the equivalent of genome average ancestry *q* [48] was lower in *V. magnifica* (7.5–7.9 %) and higher in *V. orlovi* (mean 15.5 ± 2.0 %, minimum-maximum 12.0–18.8 %). Values of inter-source population ancestry Q12 [48], which in this case is equivalent to observed heterozygosity, were lower than expected for cases of randomly mating populations or early-generation backcrosses (Fig. 1).

### Phylogenetic relationships

**Fig. 2:**
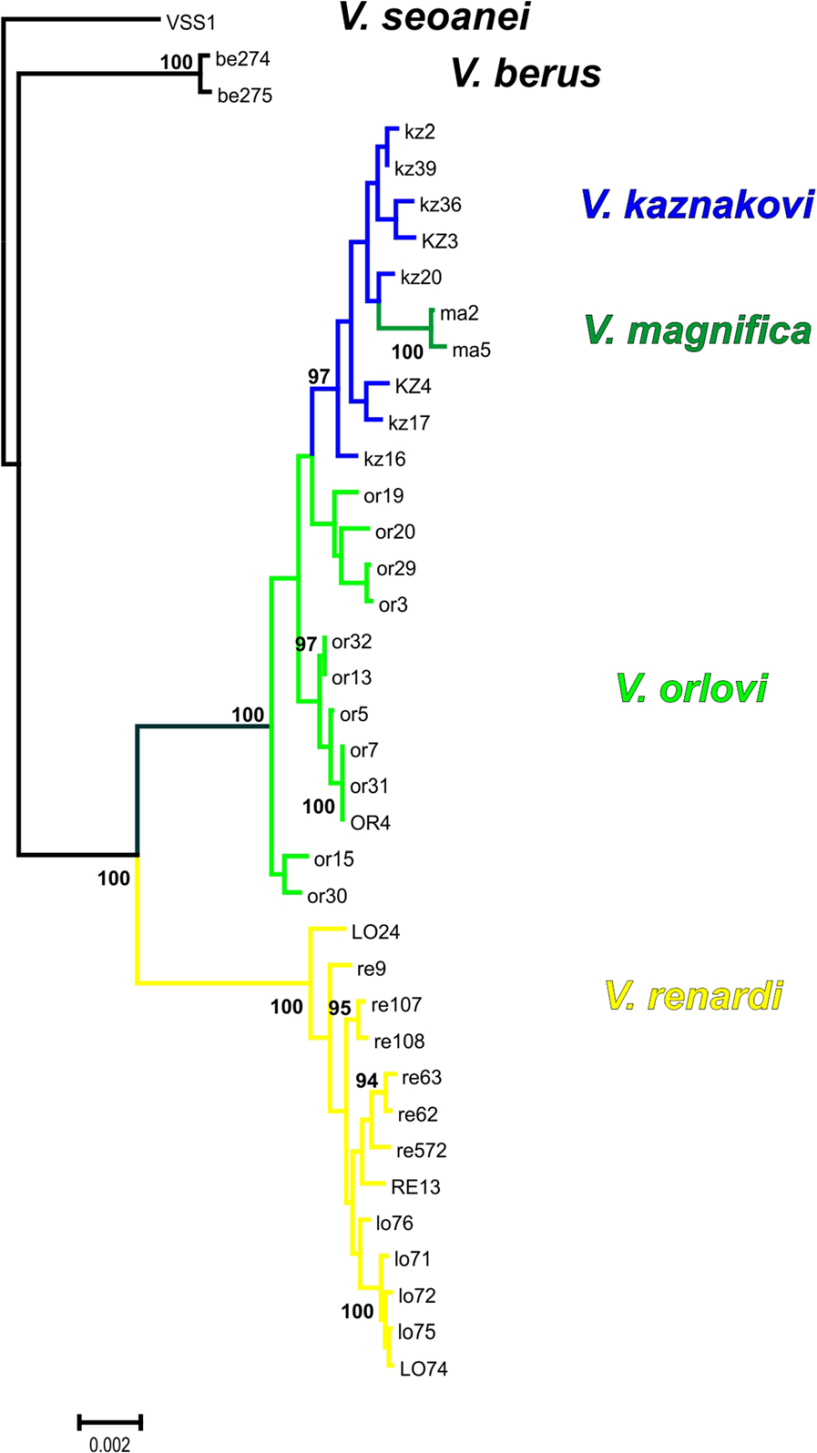
Maximum likelihood tree (RAxML v 8.2.4, 1000 bootstrap replicates) obtained from the analysis of concatenated sequence of RAD loci. Bootstrap supports above 90 % are reported at nodes

When we analysed phylogenetic relationships using Maximum Likelihood, the best supported tree showed that individuals were grouped in two main clades, with *V. berus* (Linnaeus, 1758) and *V. seoanei* Lataste, 1879 placed in the outgroup position (Fig. 2). This is similar to relationships found in previous phylogenetic analyses [26, 28, 29]. *V. orlovi* and *V. magnifica* form a well supported clade with *V. kaznakovi. V. orlovi* appears paraphyletic, with some samples basal to the rest of the clade, whereas *V. magnifica* was nested within a subclade comprised of samples of *V. kaznakovi*. All samples of *V. renardi* s. l. belong to a second large and highly supported clade, with *V. lotievi* from Dagestan in a basal position, similar to the pattern in a recent phylogenetic reconstruction based on mitochondrial DNA [29].

### Genetic cluster analyses

#### Bayesian clustering

**Fig. 3:**
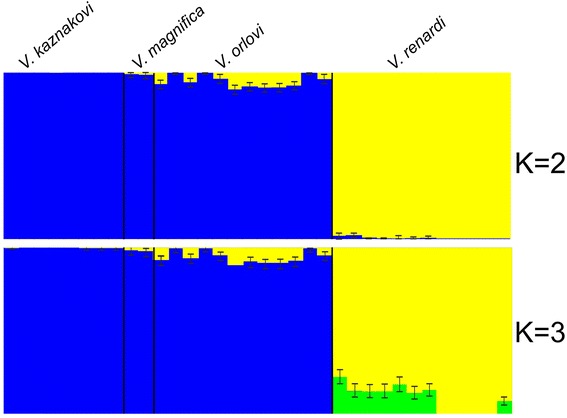
Structure plot showing assignment probabilities for each sample at K = 2. Purported hybrid taxa *V. magnifica* and *V. orlovi* are presented between the potential parental species *V. kaznakovi* and *V. renardi*

STRUCTURE identified K = 2 as the best description of genetic clusters in the data based on the maximum Delta K values in Structure Harvester. Most *V. kaznakovi* and *V. renardi* samples were assigned with nearly 100 % probability to one of the two clusters (Fig. 3). In contrast, the putative hybrid samples (*V. orlovi* and *V. magnifica*) showed evidence of relatively small but significant admixture of *V. renardi* genes into a genetic background largely characteristic of *V. kaznakovi*. Specifically, the proportion of genes characteristic of *V. renardi* in *V. orlovi* and *V. magnifica* ranged from 0.1–10.1, and the 95 % credible regions for the proportion of *V. renardi*-type genes did not overlap zero in nine out of twelve *V. orlovi* samples (Fig. 3).

Interestingly, six out of seven *V. renardi* samples from territories adjacent to the Northwestern Caucasus also exhibited a low proportion (0.2 %–2 %) of *V. kaznakovi* genes, but the 95 % confidence interval for admixture overlapped zero in all but one of these samples. In contrast, no *V. renardi* samples from the populations from Elbrus Mountain in Central Caucasus or Crimea had any traces of admixture.

#### Multivariate analysis

**Fig. 4:**
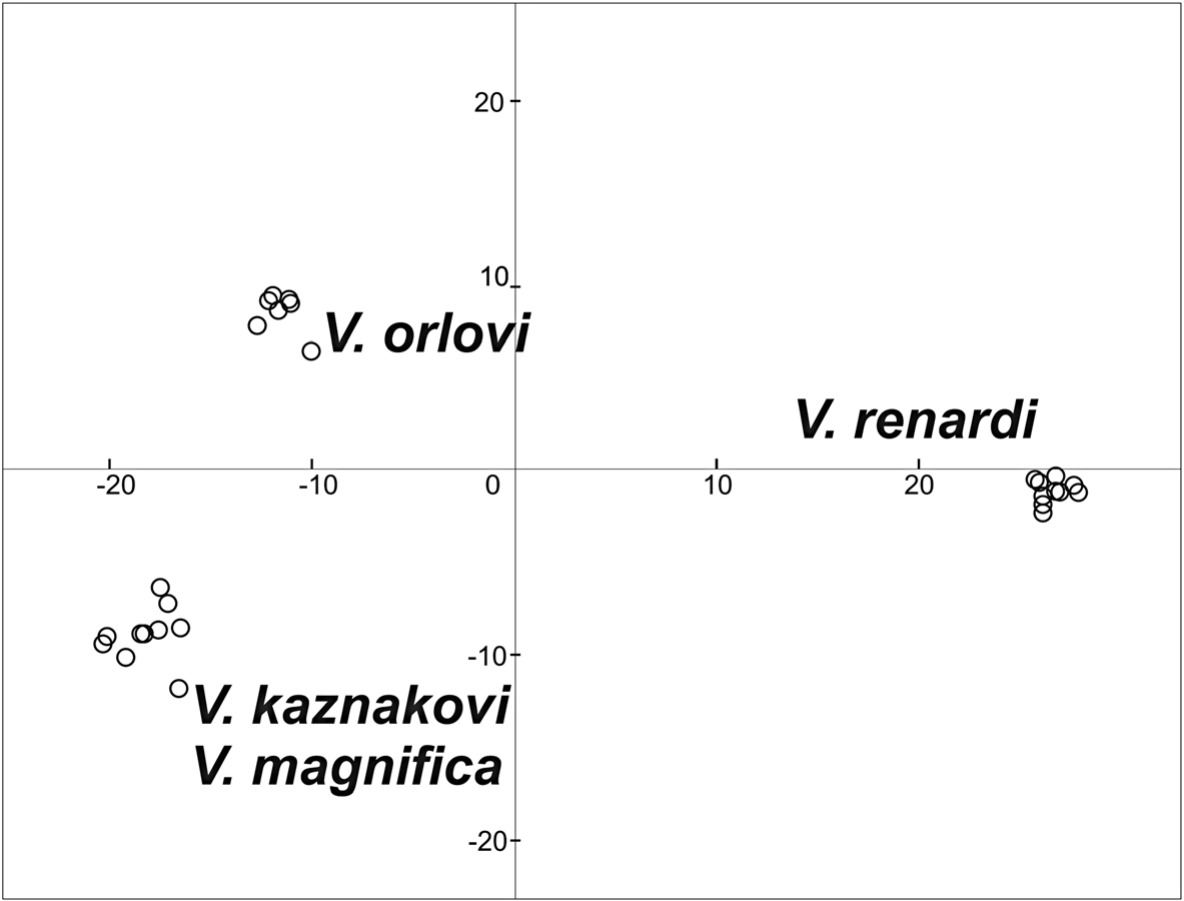
Discriminant analysis of principal components (DAPC) results for all ingroup samples. Each sample is plotted based on the first two discriminant functions. Associated clustering analyses identified three groups as the optimal solution, consisting of *V. renardi*, *V. orlovi*, and a third group containing *V. kaznakovi* and *V. magnifica*

Discriminant Analysis of Principal Components (DAPC) analysis was performed on the first eleven principal components, which accounted for 69 % of the total genetic variation. Bayesian Information Criteria (BIC) suggested that these data are best partitioned into three clusters which consisted of i) *V. renardi*, ii) *V. kaznakovi* + *V. magnifica* and iii) *V. orlovi. V. orlovi* was positioned close to the *V. kaznakovi* + *V. magnifica* group along the first two discriminant functions (DF), but was shifted towards *V. renardi* along the axis of the first DF (Fig. 4). If we measure the distance between centroids along the first-axis in percents, taking *kaznakovi*-*renardi* as 100 %, *V. orlovi* specimens had values ranging from 12.35 to 18.20 % (mean + SD: 14.87 ± 0.42) of this distance. The distance between *V. orlovi* and *V. kaznakovi* along the axis of the second DF demonstrates the presence of differentiation between these two groups. However the much lower eigenvalues of the second DF (F-statistics 1680 versus 64600) shows that magnitude of this differentiation is much lower.

In summary, results from both clustering algorithms are consistent with *V. kaznakovi* and *V. renardi* representing pure parental taxa, but these analyses did not recognize differentiation of *V. magnifica* and *V. orlovi* at a comparable level. Both of these putative hybrid groups appear to be genetically most similar to *V. kaznakovi*, but also exhibit patterns consistent with introgression of genes from *V. renardi*.

### D-statistics

**Fig. 5:**
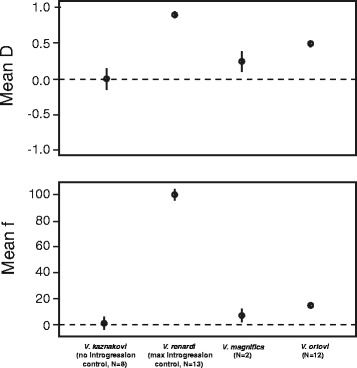
D-statistics (A) and admixture proportions, *f*, (B) estimated for each taxon included as the P2 group in ABBA/BABA tests. Nonzero D-statistics and admixture proportions indicate a history of introgression between the P2 group and *V. renardi*, which was assigned as the donor P3 group in each test. Plotted values represent means and associated 95 % confidence intervals

Individual-based tests for introgression show mean D-statistics equal to 0.24 for *V. magnifica* and 0.50 for *V. orlovi*, supporting the hypothesis of introgression for each taxon (Table 3, Fig. 5a). Associated admixture proportions (*f*) were 6.39 and 19.02 % for *V. magnifica* and *V. orlovi,* respectively (Table 3, Fig. 5b), and averages for each individual varied between 6.03 and 6.75 % in *V. magnifica* and between 14.03 and 25.40 % in *V. orlovi *(Additional file 1). As expected, control test replicates representing zero gene flow (*V. kaznakovi* as P2) produced D-statistic values close to zero on average (0, 95 % CI -0.15–0.15), while those representing maximal gene flow (*V. renardi* as P2), produced high mean D-statistic values (0.91, 95 % CI 0.90–0.92, Fig. 5a; Additional file 1). D-statistics calculated with the population-based method were similar to those in the individual based tests (0.26 and 0.53 for *V. magnifica* and *V. orlovi*, respectively), and the range of D-statistics from resampled datasets did not include zero for either group (Table 3).

**Table 3:** Results of Patterson’s D-statistic test for taxa of putative hybrid origin (*V. magnifica*, *V. orlovi*)

Group	Individual based calculations					Population based calculations	
	Number of tests, N	Patterson D, averaged across all tests	Number of ABBA sites, averaged across all tests	Number of BABA sites, averaged across all tests	Admixture proportion, f, %	Patterson D	Number of ABBA and BABA sites
*V. magnifica*	9968	0.24 ± 0.23	9.71 ± 2.60	5.99 ± 2.40	6.39 ± 6.05	0.26 ± 0.08	77
		-0.57–0.86	2–20	1–16	-13.74–22.33	0.08–0.45	
*V. orlovi*	59859	0.50 ± 0.18	16.63 ± 3.43	5.56 ± 2.30	19.02 ± 7.24	0.53 ± 0.03	135
		0.29–1.0	5–29	0–16	-8.59–41.23	0.43–0.60	

Statistics for individual-based tests are reported as the mean ± SD and range calculated across all individuals evaluated as the P2 taxon. For these tests, the P1 taxon is always represented by *V. kaznakovi*, and the P3 taxon is always represented by *V. renardi*. Values reported for the population-based analyses represent the point estimate of D, and the range of D obtained from 100 resampled datasets

### Effects of variability in P1, P3 and outgroup samples on D-statistics

To evaluate the effect that variation among samples representing the P1, P3, and the Outgroup taxa may have on D-statistics, we tested for bias in D-statistics associated with including individual samples in P1, P3, and the Outgroup taxa. Specifically, we obtained D-statistics for all runs that contained a given sample in one of these groups. We then tested whether any of the samples representing P1, P3, or the Outgroup samples were associated with biased D-statistics. The choice of individual in each reference group (P1, P3 and the Outgroup) had a significant effect on D (*p* < 0.00001), though the overall magnitude of the mean differences was generally low and did not exceed 0.03 for the Outgroup specimens, and 0.09 and 0.08 for P1 and P3 samples, respectively. However, two notable outliers were *V. kaznakovi* individuals Kaz17 and Kaz3 in the P1 group, which increase this difference to 0.2939 and 0.1775, respectively, demonstrating that specimen choice can have substantial effects on the signal of introgression.

## Discussion

### Evaluation of introgression in Caucasus vipers

Analyses of RAD loci using both clustering methods, explicit tests for introgression based on Patterson’s D-statistic and direct calculation of genome average ancestry using loci fixed for different SNP (Single-nucleotide Polymorphism) alleles between the two parental species all support the hypothesis that genetic introgression has influenced the evolution of the currently-described viper species *V. orlovi* and *V. magnifica*. The variation in terms of patterns of heterozygosity and Fst values are also consistent with introgression leading to the emergence of hybrid populations of these two taxa.

Previous studies have demonstrated how clustering methods and more direct approaches such as ABBA/BABA tests can complement each other in testing for historical introgression [i.e. 44]. For example, clustering methods have the advantage of allowing an exploration of the data without requiring any a priori assignment of samples to groups. However, confidently inferring introgression directly from these clustering methods can be difficult, as any patterns of genetic admixture produced from these methods can also arise from incomplete lineage sorting. ABBA/BABA tests provide an explicit way to distinguish between incomplete lineage sorting and introgression, but require *a priori* hypotheses about patterns of hybridization.

One drawback to ABBA/BABA tests is that nucleotide site patterns useful for this test are uncommon in the genome, and even with genomic-scale SNP data, the number of ABBA/BABA sites for a test can be limited. In these cases, statistical power for inferring historical introgression on a sample-by-sample basis can be low (often measured with Z-statistics that are based on bootstrap resampling of loci). In previous studies, when the number of sites was sufficient, the authors did not include sites which were heterozygous [41]. In contrast we used both alleles at such sites so as not to lose any information, ran all possible combinations of the test, and summarized the results by averaging them. This approach both increased the number of informative loci, and allowed for an evaluation of the impact of variation among samples on D-statistic results.

Patterson’s D-statistic is calculated for four individual sequences at a time. When many individuals from each of the four groups are sequenced, and there is variation in both parental populations due to potential presence of admixed individuals, the explicit calculations of D-statistics for all possible combinations of sequences is the only way to avoid bias. Our analysis showed that for tests in which two specific *V. kaznakovi* samples were included as the P1 taxon, the average D-statistics over all P2 samples analyzed were biased as compared to all of the remaining P1, P3, and the Outgroup sample combinations. The specific reason for this bias is not clear, but it could be due to the use of introgressed specimens or geographically isolated subpopulations of *V. kaznakovi* themselves. For example, sample Kaz17, when tested as a possible hybrid (assigned as the P2 taxon in the ABBA/BABA test) has a mean D higher than *V. magnifica* (0.35 ± 0.24) (Additional file 1). This result suggests that when possible, D-statistic tests should be evaluated with multiple samples representing each taxon in order to account for any important variability introduced with different samples representing the P1, P3, and the Outgroup categories. While population-based calculations of D-statistics are less susceptible to stochasticity when a low number of informative sites are available, they have the disadvantage of potentially overlooking introgressed individuals assigned to P1 and P3 populations.

Durand et al. [46] and Eriksson and Manica [49] point to ancient subdivision as a possible source of inflated D-statistics. We feel this is not an issue with our data set: other analyses do not support presence of long-lasting subdivisions of viper populations in Caucasus: *V. orlovi* and *V. magnifica* do not form separate clusters (Fig. 3) or monophyletic clades (Fig. 2) which would be expected if they had been isolated from *V. kaznakovi* for considerable time. In addition, due to its northern position and the Pleistocene climatic fluctuations in this part of the Caucasus region, it seems unlikely that stable long term environmental barriers could exist leading to subdivided populations.

Both proposed hybrid taxa have similar magnitudes of Fst values when compared to each of the presumed parental species: low when compared to *V. kaznakovi* and high in comparisons with *V. renardi*. According to Fst values, *V. magnifica* is more similar to *V. kaznakovi* then *V. orlovi* (Table 2). At the same time they both have a low number of private alleles, (Table 1). Higher values of overall Ho in these two groups is expected under a scenario of hybridization. However, Ho is lower than He in *V. renardi*, which indicates possible inbreeding in this group.

### Biology and ecology of viper hybridization

Most species of small vipers have allopatric distributions, and species complexes are ecologically linked to different habitat types, which together with geographic isolation lead to the development of reproductive barriers between species [23, 24, 32, 33]. There are only a few documented cases in which two or more genetically and ecologically distinct species live in sympatry, isolated by habitat preferences and altitude. These include *V. berus* and *V. ursinii* (Bonaparte, 1835) in Southern Europe [25], *V. berus* s. l. and *V. renardi* in Eastern Europe [51, personal observations], *V. dinniki* and *V. lotievi* Nilson et al. 1995 in Northern Caucasus [52], *V. darevskii* and *V. eriwanensis* in Transcaucasia [23], and *V. ammodytes* (Linnaeus, 1758), *V. aspis* (Linnaeus, 1758) and *V. berus* in Slovenia [53]. The contact and possible coexistence of species in an ecotone has been documented between the closely related viper species *V. aspis*, *V. latastei*Boscá, 1878, and the more distantly related *V. seoanei* in Spain [22], where transition areas between different habitat types are associated with random hybridization between the vipers inhabiting them. The ecological differentiation and microhabitat segregation in this case prevents high levels of gene flow [22].

Though the area occupied by *V. orlovi* covers a considerable geographic range, no migrants representing pure parental species were found in sympatry with the hybrids, and are unlikely to be present due to the size of the range of *V. orlovi* compared to the activity range of individual snakes in vipers. In addition to this, several lines of genetic evidence argue against the occurrence of ongoing gene flow. For example, there was no pronounced clinal variation in the admixture proportion across *V. orlovi* localities, and also no evidence of a hybrid swarm in either *V. orlovi* or *V. magnifica* (Additional file 1, Fig. 3,). Instead, there are rather uniform admixture proportions within both *V. orlovi* and *V. magnifica* samples, indicating little or no current hybridization with parental species (though there are traces of introgression in marginal populations of *V. renardi* and *V. kazankovi* and the admixture proportions between the two hybrid groups did differ, with *V. orlovi* displaying more admixture than *V. magnifica* – Table 3, Fig. 5). In addition, low means of an inter-source population ancestry Q12 in *V. magnifica* or *V. orlovi* indicates that in our dataset none of individuals represented backcrosses where at least one of the parents was pure individual of *V. kaznakovi* or *V. renardi*.

In contrast to ongoing hybridization, these data broadly point to historical hybridization and introgression as factors in the evolution of *V. orlovi* and *V. magnifica*. Paleogeographic reconstructions [54] indicate a complex history involving numerous fluctuations between open and forest habitat type in the northwestern Caucasus throughout the Quaternary. This ecological instability, coupled with the proximity of northwestern Caucasus forests to the steppes of Cis-Caucasia, may have facilitated contact between the two species complexes (*V. renardi* and *V. kaznakovi*), resulting in hybridization, and/or may have established novel niches where hybrid specimens are more fit compared to parental individuals [1].

Though it appears to have happened in the past, the specific timescale for the hybridization is not clear from the data presented here. The earliest time at which hybridization likely could have occurred coincides with the time when steppe vipers first colonized the Northern Caucasus region, which was recently estimated at approximately 380,000 ybp [29]. Nevertheless, more recent hybridization is also a possibility, potentially coinciding with anthropogenic effects that have influenced habitat and reduced the effectiveness of ecological reproductive barriers. Hybridization may have resulted from a range expansion by *V. renardi* that was associated with increases in secondary open grasslands (pastures) after anthropogenic deforestation of edge territories along the range of *V. kaznakovi*. Consequently, after the displacement and expulsion of local Circassians (Adygs) by the Russian Empire in 1864 [55], forests started to recover and open grasslands had eventually declined [56], which may have resulted in the current distributions and lack of ongoing gene flow between *V. renardi* and *V. kaznakovi*. Indeed, the habitat occupied by *V. orlovi* and *V. magnifica* in the northwestern Caucasus is a mosaic of drier sub-Mediterranean xerophyte forests, meadows, and mountain steppes that has experienced significant anthropogenic impacts, and has undergone succession after a change in the prevailing type of human activity over the past two centuries [56]. Tendency towards Ho excess over He, higher allelic richness, and lower numbers of private alleles (Table 1) could be viewed as evidence consistent with more recent admixture, but without knowing baseline of these values under different historical scenarios involving hybridization we cannot draw any conclusions about the timing of admixture. Further analyses such as historical demographic modelling may help to better assess the timescale of these hybridization events.

In summary, we propose that human mediated habitat transformation in the contact zone between distant species of vipers may have resulted in novel habitats differing from those of the parental species that were colonized by a stabilized hybrid population. This population of *V. orlovi* which has originated through hybridization may possess a specific combination of parental genetic features that make it more fit in the particular environmental conditions. If this assumption is confirmed by additional ecological data and evidence of non-random introgression and fixation of adaptive alleles (adaptive divergence initiated by hybridization – [57]), *V. orlovi* may serve as an example of recent hybrid speciation initiated by habitat disturbance.

### Taxonomic and conservation issues

*V. orlovi* is a critically endangered species according to the IUCN classification [32] and *V. magnifica* is listed as an endangered species [33], with both showing evidence of declining populations in nature. However, our results indicate that both have hybrid ancestry and are not distinct monophyletic lineages, and therefore cannot be described as taxa according to The International Code of Zoological Nomenclature [58]. In turn, because they are not species or subspecies, they should be excluded from the IUCN Red list. Instead both *V. orlovi* and *V. magnifica* may be listed as regional populations within *V. kaznakovi*, with which they share the most genetic material and contain a considerable part of the genetic diversity of this species [59, 60]. At the same time, the hybrid populations between distinct lineages of small vipers that occupy habitats unique from the parental species are important for our understanding of evolution and speciation and should be conserved as a unique natural phenomenon and treated as distinct evolutionary entities deserving conservation attention [61]. If we further assume that hybrid speciation may have occurred in this particular case of *V. orlovi,* it should be protected; this would mean that it may be recognized as a taxon of hybrid origin and that the official recommendations of The International Code of Zoological Nomenclature regarding hybrids must be changed, as well as the more general view of species as monophyletic groups in animals. However, *V. magnifica* has a more restricted distribution, a marginal signal of admixture, and non-distinct habitat differences, making this taxon equivalent to isolated *V. kaznakovi* populations, and so we believe it therefore should be synonymised with the latter.

## Conclusions

According to our results, a hybridization event between *V. kaznakovi* and *V. renardi* contributed to genetic diversity that currently characterizes *V. orlovi*, which occurs in an intermediate habitat type relative to the two parental species. Contact zones between grasslands, which are typical habitats of *V. renardi*, and humid forest, which are typical habitats of *V. kaznakovi*, and/or human-mediated environmental disturbance, may have facilitated the formation of this hybrid group. Relative to *V. orlovi*, a smaller amount of introgression was detected in *V. magnifica.* Given this result, and an absence of ecological specialization associated with *V. magnifica*, we suggest that from both a taxonomic and conservation perspective it may best be treated as a marginal population of *V. kaznakovi*.

## Methods

### Sampling

We sampled tissues from ethanol-preserved museum collection or dry shed skin obtained from alive snakes after shedding in a research collection of Tula Exotarium (Additional file 2). No additional fieldwork or experiments were carried out with snakes in nature or captivity.

**Fig. 6:**
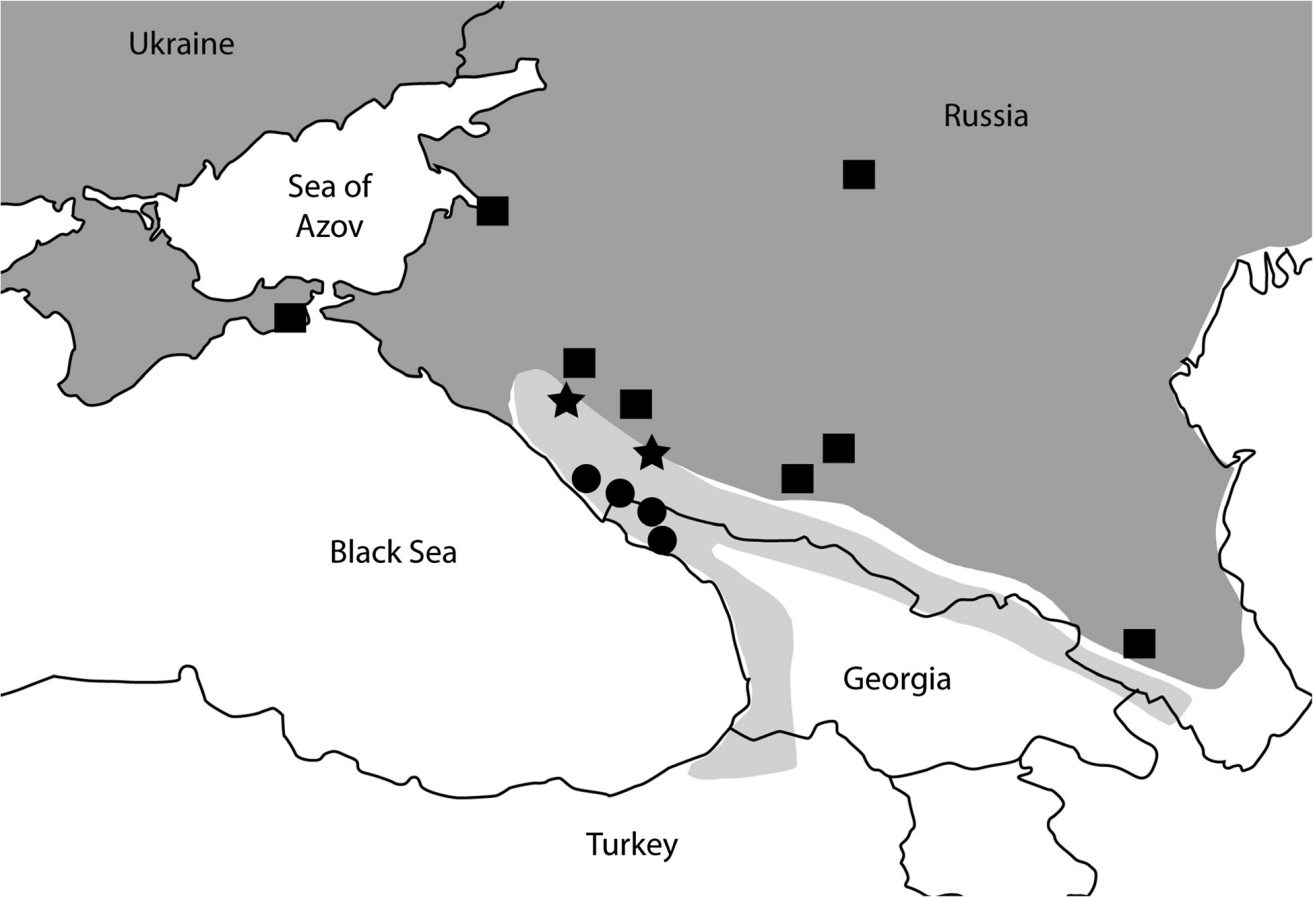
Approximate sampling localities of *V. renardi* (squares), *V. kaznakovi* (circles), and potential hybrid taxa *V. orlovi* (western star) and *V. magnifica* (eastern star). Distributions of the *V. renardi* and *V. kaznakovi* complexes are shown for Eastern Europe and Caucasus in dark and light gray, respectively.

Samples within the *V. kaznakovi* complex (*V. kaznakovi* and potential hybrid taxa *V. orlovi* and *V. magnifica*) were assigned to taxa based on previously described species ranges, as reliable diagnostic morphological characters are not available [30, Additional file 2]. *V. kaznakovi* samples (*N* = 8) were from low-altitude forest habitats in northwestern Caucasus just to the southeast of the range of *V. orlovi* (Fig. 6). Specimens of *V. orlovi* (*N* = 12) came from south of the type locality, within the area of the Western Caucasus occupied by *V. orlovi* according to the original description [30]. Specimens of *V. magnifica* (*N* = 2) belong to the type series (holotype and paratype) from the Sochi National Park Museum collection.

*V. renardi* s. l., including *V. lotievi* sensu Tuniyev et al. [50], could be easily differentiated from all species of the *V. kaznakovi* complex, as it typically has one apical shield and a sharp snout [25]. *V. renardi* samples (*N* = 13) were collected from populations in Southern Russia and Ukraine, including five specimens of *V. lotievi* from Elbrus Mountain in Central Caucasus and one specimen from Dagestan in Eastern Caucasus (Fig. 6, Additional file 2). Samples of *V. berus* (from Russia, *N* = 2) and *V. seoanei* (from Spain, *N* = 1), which occur in a clade sister to the clade containing *V. renardi* and *V. kaznakovi* [26, 28], were used as outgroup samples in the analyses.

### Genetic analyses

DNA was extracted from blood, scale clips, shed skin, or ethanol fixed tissue using DNeasy Blood & Tissue Kits (Qiagen). DNA concentrations were measured on a Qubit 2.0 fluorometer (Invitrogen) and adjusted to a working concentration of 15 ng/uL.

Double-digest RADSeq libraries were generated with the protocol described in Sovic et al. [62]. Briefly, genomic DNA was digested with EcoRI and SbfI restriction enzymes and barcoded adaptors were ligated onto the digestion products from each sample. Fragments of size 300–450 bp were then selected using gel electrophoresis, amplified using Polymerase Chain Reaction (PCR), and purified with streptavidin beads. Products were quantified by qPCR (Quantitative real-time PCR) using KAPA library quantification kits after 1:1500 dilution, and these qPCR measurements were used to pool individuals in equimolar concentrations for sequencing. Pooled libraries were run in single read 50-bp runs on an Illumina sequencer. SNP identification and genotyping were performed using AftrRAD version 3.3 [63] with the following parameters: Phred quality scores had to exceed 20 to retain the base call, minimum average read depth was 20 per allele, reads were considered allelic if they exhibited at least 90 % sequence similarity, and paralogous loci were identified as loci with a minimum of 10 reads at a third allele in at least one individual. We retained for analysis only loci genotyped in all samples in the dataset.

### Data analyses

#### Descriptive statistics of genetic variation

Basic genetic statistics and pairwise Fst values was calculated using GENEPOP and Arelquin software [64, 65].

To assess whether presumably hybrid specimens appear to result from ongoing hybridization, we applied a method patterned after that of Gompert et al. [48]. Specifically, we used a subsample of the dataset that included biallelic loci with different alleles fixed in *V. kaznakovi* and *V. renardi.* The admixture proportion *q* (similar to those obtained in STRUCTURE) was directly calculated from these allele frequencies in each individual. Knowing the admixture proportion *q* we can calculate the proportion of heterozygous loci (denoted as Q12 or inter-source population ancestry [48]). The maximum value of Q12 will occur in a case of ongoing hybridization when one of the parents is a pure parental individual. In the case of randomly mating hybrid population, Q12 will be the expected value of heterozygosity under Hardy-Weinberg equilibrium.

### Phylogenetic analysis

We constructed a Maximum Likelihood phylogenetic tree using RAxML v 8.2.4 [66] using all concatenated loci and GTRCAT approximation of models for 1000 bootstrap replicates.

### Genetic clustering

Genetic clustering was performed on 34 samples (excluding outgroup taxa and one sample of *V. lotievi* from Dagestan because the entire separate clade of *V. lotievi* from Dagestan [29] was represented by only one specimen) to evaluate potential genetic admixture using two different approaches. The first was STRUCTURE [67], which uses Bayesian methods to optimally cluster individuals under a simple admixture model by maximizing Hardy-Weinberg equilibrium within groups. We also clustered samples by Discriminant Analysis of Principal Components (DAPC), which uses a k-means clustering algorithm to identify groups rather than assuming Hardy-Weinberg equilibrium within groups, as implemented in the R package Adegenet 1.3–9.2 [68, 69]. Both methods can evaluate the amount of admixture of individuals either by calculating the posterior probability of assignment to parental populations (Structure) or by projecting individuals from parental and hybrid populations along axes of discriminant function scores (DAPC).

Structure was run for K values ranging from one to six, with three independent runs at each K. Each run consisted of 200000 MCMC iterations, after an additional burnin period of 20000, and allowed for admixture and correlated allele frequencies. The optimal number of clusters (K) was identified using Structure Harvester [70]. No prior information about group assignment of individuals was provided.

For DAPC runs, the number of retained principal components was chosen based on the cumulative variance explained. The optimal number of clusters was then identified using Bayesian information criteria [71].

### D Statistics

**Fig. 7:**
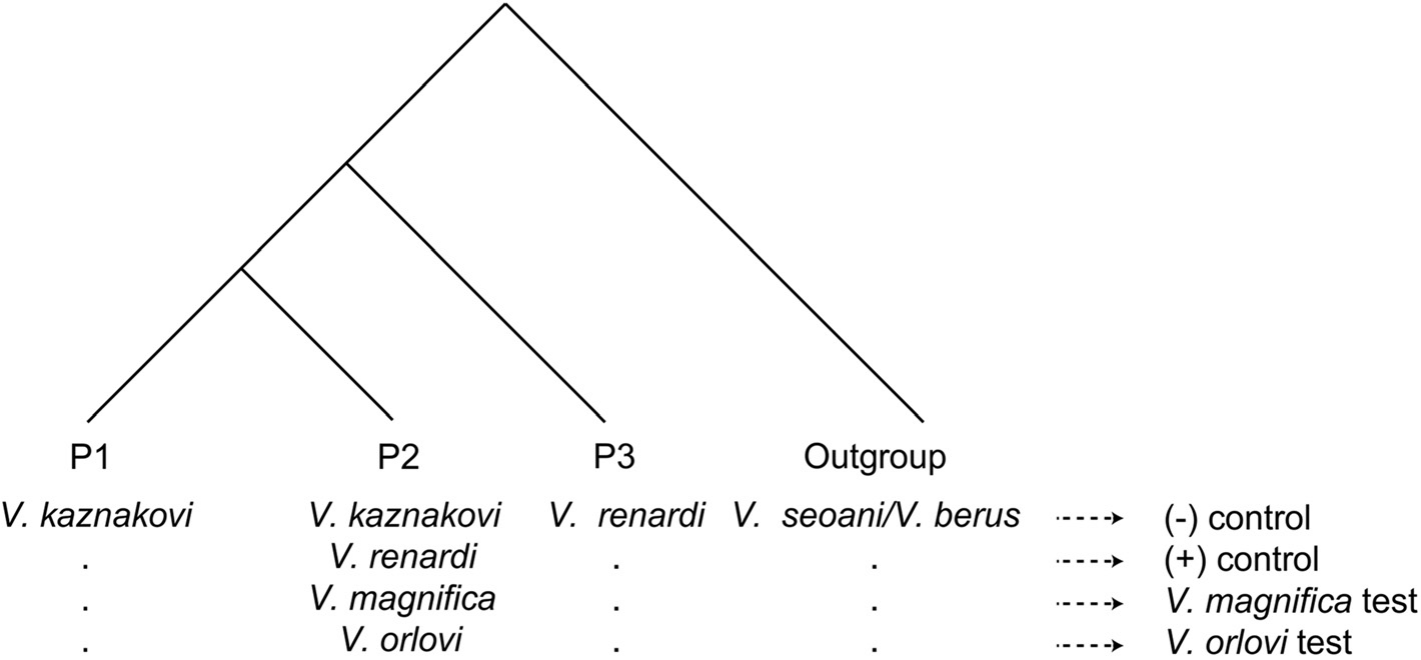
Various arrangements of taxa used for ABBA/BABA tests to calculate D-statistics. Gene flow from the P3 group (*V. renardi* in all cases) into the P2 group is expected to increase the number of sites displaying the ABBA pattern relative to the BABA pattern, pushing the D statistic toward 1

A weakness of clustering approaches in hybridization studies is that they do not account for incomplete lineage sorting (ILS) as a cause of similarity between groups. Therefore we also applied the Patterson D-statistic (ABBA/BABA) test [46] to more explicitly evaluate potential genetic introgression within these vipers. This test takes advantage of genomic-scale data by evaluating the subset of biallelic loci that produce incongruent gene trees with the pattern ABBA or BABA on a species tree (((P1,P2),P3),P4), where taxon P2 is a putative hybrid taxon, taxa P1 and P3 are potential parental taxa, and taxon P4 is an Outgroup (Fig. 7). In the absence of introgression, gene trees with the ABBA and BABA patterns are expected to occur in equal frequencies, while introgression causes deviations from a 1:1 ratio of these patterns. These deviations are measured by Patterson’s D, which approaches 1 if gene flow occurs from P3 to P2 [41, 43, 45, 46, 72, 73].

We assigned *V. kaznakovi* and *V. renardi* as the P1 and P3 groups, respectively, and tested for evidence of gene flow from *V. renardi* into each of the purported hybrid groups, *V. orlovi* and *V. magnifica* (P2, Fig. 7)*.* We also calculated D statistics with *V. kaznakovi* and *V. renardi* as the P2 taxa, which served as controls representing minimum (*V. kaznakovi*) and maximum (*V. renardi*) levels of introgression. The tests in which *V. renardi* was included as the P2 group were also used to obtain the maximum potential number of introgressed sites for calculations of conservative minimum estimates of the admixture proportion *f* for *V. magnifica* and *V. orlovi*, according to equation 8 of Durand et al. [46].

Because we had multiple samples representing each of our taxa, we explored the impact that individual variation had on values of D by generating D-statistics for all possible combinations of individuals representing the P1, P3, and Outgroup taxa for each P2 sample tested. Each combination was run twice to account for both alleles in the diploid samples. Mean D and *f* statistics were obtained for each sample as the average over all runs for which the specific sample was included as the P2 taxon. This approach utilizes all of the information in the data, in contrast to previous treatments, which have either excluded all heterozygous loci from the analyses [41] or randomly taken one of two alleles from diploid samples [47]. The mean D and *f* statistics were then averaged over all samples representing each P2 taxon or for each individual to draw inference regarding the occurrence of introgression, and to estimate admixture proportion. We used combination of dstat.R script (https://gist.github.com/coleoguy/6886683) modified by us and two Perl scripts to automate the process of generation of alignments from individual sequences and calculation of D statistic for each of these alignments.

The D statistic was also calculated from population allele frequencies according to Equation 2 in Durand et al [46]. The derived allele at each locus was defined as the least frequent allele in the Outgroup. The significance of the observed D statistic was assessed by calculating D statistics from 100 bootstrapped datasets in which derived allele frequencies were resampled from a binomial distribution. This distribution was specified by the total number of alleles sampled in the original dataset and the observed frequency of the derived allele at the relevant locus/population combination.

### Availability of data and materials

Raw Illumina reads of ddRAD libraries, barcode information and scripts to calculate individual and population D statistic are available from the Dryad Digital Repository: doi:10.5061/dryad.c5232.

### Ethics

Not applicable.

### Consent to publish

Not applicable.

## Additional files

Additional file 1: D-test and admixture proportion *f* [46] for *V. kaznakovi*, *V. renardi* (including *V. lotievi*), *V. orlovi,* and *V. magnifica*. Each row represents data from all combinations of possible P1, P3, and the Outgroup individuals, where P1 is always represented by *V. kaznakovi* and P3 is always represented by *V. renardi.* (DOC 96 kb) Additional file 2: List of samples used with information about species, locality, date of collection, collector, and institution where collections are stored. (XLS 34 kb)

## References

[1] Arnold ML (1997). Natural Hybridization and Evolution.

[2] Mallet J (2005). Hybridization as an invasion of the genome. Trends in Ecology and Evolution.

[3] Seehausen O, Takimoto G, Roy D, Jokela J (2008). Speciation reversal and biordiversity dynamics with hybridization in changing environments. Mol Ecol.

[4] Sanders KL, Rasmussen AR, Guinea ML (2014). High rates of hybridisation reveal fragile reproductive barriers between endangered Australian sea snakes. Biol Conserv.

[5] Harvey MB, Barker DG, Ammerman LK, Chippindale PT (2000). Systematics of pythons of the *Morelia amethistina* complex (Serpentes: Boidae) with the description of three new species. Herpetol Monogr.

[6] Fitzpatrick BM, Placyk JR, Niemiller ML, Casper GS, Burghardt GM (2008). Distinctiveness in the face of gene flow: hybridization between specialist and generalist gartersnakes. Mol Ecol.

[7] Mebert K (2008). Good species despite massive hybridization: genetic research on the contact zone between the *watersnakes Nerodia sipedon* and *N. fasciata* in the Carolinas, USA. Mol Ecol.

[8] LeClere JB, Hoaglund EP, Scharosch J, Smith CE, Gamble T (2012). Naturally occuring intergeneric hybrid snakes (*Pituophis catenifer sayi* x *Pantherophis vulpinus*; Lampropeltini, Squamata) from the Midwestern United States. J Herpetol.

[9] Placyk JS, Fitzpatrick BM, Casper GS, Small RL, Reynolds RG, Noble DWA, Brooks RJ, Burghardt GM (2012). Hybridization between two gartersnake species (*Thamnophis*) of conservation concern: a threat or an important natural interaction?. Conserv Genet.

[10] Vandewege MW, Rodriguez D, Weaver JP, Hibbitts TD, Forstner MRJ, Densmore LD (2012). Evidence of Hybridization between *Elaphe bairdi* and *Elaphe obsoleta lindheimeri* Including Comparative Population Genetics Inferred from Microsatellites and Mitochondrial DNA Source. J Herpetol.

[11] Kapfer JM, Sloss BL, Schuurman GW, Paloski RA, Lorch JM (2013). Evidence of Hybridization between Common Gartersnakes (*Thamnophis sirtalis*) and Butler’s Gartersnakes (*Thamnophis butleri*) in Wisconsin, USA. J Herpetol.

[12] McDiarmid RW, Campbell JA, Touré TA (1999). Snake species of the world. Vol. 1. Herpetologists’ League.

[13] Saint GH (1990). Morphologie comparée des hybrides de *Vipera seoanei* LATASTE, 1879 x *Vipera aspis* (L.). Amphibia-Reptilia.

[14] Saint GH (1990). Croissance, maturite sexuelle et variations ontogeniques des periodes d’alimentation et des mues chez deux viperes hybrides (*Vipera aspis* x *Vipera seoanei*) dans des conditions semi-naturelles. Bulletin de la Societe Herpetologique de France.

[15] Venczel M, Ghira I (1994). A preliminary study on the osteology of nose-horned viper (Vipera ammodytes ammodytes L. 1758) from Boiu de Sus, Romania. “Sargetia”. Series Sciencia Naturae.

[16] Naullau G (1997). La Vipere Aspic.

[17] Ispas G, Ghira I, Rakosy L, Kirsch M (2000). An improved method to analyze snakes chromosome complement. Evolution and Adaptation.

[18] Guillemin I, Bouchier C, Garrigues T, Wisner A, Choumet V (2003). Sequences and structural organization of phospholipase A2 genes from *Vipera aspis aspis*, *V. aspis zinnikeri* and *Vipera berus berus* venom Identification of the origin of a new viper population based on ammodytin I1 heterogeneity. Eur J Biochem.

[19] Zinenko OI (2004). New data about hybridization between *Vipera nikolskii* Vedmederya, Grubant et Rudaeva, 1986 and *Vipera berus berus* (Linnaeus, 1758) and their contact zones in Ukraine. Mertensiella.

[20] Shiryaev KA, Ananjeva N, Tsinenko O (2005). New data on reproductive biology of Caucasian species of the genus *Vipera*.

[21] Pavlov AV, Zinenko OI, Joger U, Stümpel N, Petrova IV, Malenyov AL, Zaitseva OV, Shurshina IV, Bakiev AG (2011). Natural hybridization of the Eastern Steppe Viper *Vipera renardi* and the Common Adder *V. berus*. Proceedings of Samara Scientific Center of the Russian Academy of Sciences.

[22] Tarroso P, Pereira RJ, Martínez-Freiría F, Godinho R, Brito JC (2014). Hybridization at an ecotone: ecological and genetic barriers between three Iberian vipers. Mol Ecol.

[23] Orlov N, Tuniyev B (1990). Three Species in the *Vipera kaznakovi* Complex (Eurosiberian Group) in the Caucasus: Their Present Distribution, Possible Genesis, and Phylogeny. Asiatic Herpetological Research.

[24] Saint Girons H (1980). Biogéographie et évolution des vipéres européennes. CR Soc Biogeogr.

[25] Nilson G, Andrén C (2001). The meadow and steppe vipers of Europe and Asia – the *Vipera (Acridophaga) ursinii* complex. Acta Zool Acad Scien Hung.

[26] Kalyabina-Hauf S, Schweiger S, Joger U, Mayer W, Orlov N, Wink M (2004). Phylogeny and systematics of adders (*Vipera berus* complex). Mertensiella.

[27] Joger U, Fritz U, Guicking D, Kalyabina-Hauf S, Nagy ZT, Wink M (2007). Phylogeography of western Palaearctic reptiles – Spatial and temporal speciation patterns. Zool Anzeiger.

[28] Ferchaud A-L, Ursenbacher S, Cheylan M, Luiselli L, Jelic D, Halpern B, Major A, Kotenko T, Keyans N, Behrooz R, Crnobrnja-Isailovic J, Tomovic L, Ghira I, Ioannidis Y, Arnal V, Montgelard C (2012). Phylogeography of the *Vipera ursinii* complex (Viperidae): mitochondrial markers reveal an east–west disjunction in the Palaearctic region. J Biogeogr.

[29] Zinenko O, Stümpel N, Mazanaeva L, Bakiev A, Shiryaev K, Pavlov A (2015). Mitochondrial phylogeny shows multiply independent ecological transitions and northern dispersion despite of Pleistocene glaciations in meadow and steppe vipers (*Vipera ursinii* and *Vipera renardi*). Mol Phylogenet Evol.

[30] Tuniyev BS, Ostrovskikh SV (2001). Two new species of vipers of “kaznakovi” complex (Ophidia, Viperidae) from western Caucasus. Russ J Herpetol.

[31] Zamotailov AS, editor. The Red Data Book of Krasnodar region (Animals). 2nd ed. Krasnodar: Dizain Buro 1; 2007.

[32] Tuniyev B, Nilson G, Agasyan A, Orlov N, Tuniyev S. Vipera orlovi. In: The IUCN Red List of Threatened Species. Version 2014.2. 2009. www.iucnredlist.org. Accessed 24 July 2014.

[33] Tuniyev B, Nilson G, Agasyan A, Orlov N, Tuniyev S. Vipera magnifica. In: The IUCN Red List of Threatened Species. Version 2014.2. 2009. www.iucnredlist.org. Accessed 24 July 2014.

[34] Sunnucks P (2000). Efficient genetic markers for population biology. TREE.

[35] Liu ZJ, Cordes JF (2004). DNA marker technologies and their applications in aquaculture genetics. Aquaculture.

[36] Brewer MS, Cotoras DD, Croucher PJP, Gillespie RG (2014). New sequencing technologies, the development of genomics tools, and their applications in evolutionary arachnology. J Arachnol.

[37] Sovic M, Kubatko LS, Fuerst PA (2014). The effects of locus number, genetic divergence, and genotyping error on the utility of dominant markers for hybrid identification. Ecology and Evolution.

[38] Davey JW, Hohenlohe PA, Etter PD, Boone JQ, Catchen JM, Blaxter ML (2011). Genome-wide genetic marker discovery and genotyping using next-generation sequencing. Nat Rev Genet.

[39] Hohenlohe PA, Bassham S, Etter PD, Stiffler N, Johnson EA, Cresko WA (2010). Population genomics of parallel adaptation in threespine stickleback using sequenced RAD tags. PLoS Genet.

[40] Hohenlohe PA, Amish SJ, Catchen JM, Allendorf FW, Luikart G (2011). Next-generation RAD sequencing identifies thousands of SNPs for assessing hybridization between rainbow and westslope cutthroat trout. Mol Ecol Resour.

[41] Eaton D, Ree R (2013). Inferring Phylogeny and Introgression using RADseq Data: An Example from Flowering Plants (*Pedicularis*: Orobanchaceae). Syst Biol.

[42] Cruaud A, Gautier M, Galan M, Foucaud J, Saune L, Genson G, Dubois E, Nidelet S, Deuve T, Rasplus J-Y (2014). Empirical Assessment of RAD Sequencing for Interspecific Phylogeny. Mol Biol Evol.

[43] Rheindt FE, Fujita MK, Wilton PR, Edwards SV (2014). Introgression and phenotypic assimilation in *Zimmerius* Flycatchers (Tyrannidae): population genetic and phylogenetic inferences from genome-wide SNPs. Syst Biol.

[44] Streicher JW, Devitt TJ, Goldberg CS, Malone JH, Blackmon H, Fujita MK (2014). Diversification and asymmetrical gene flow across time and space: lineage sorting and hybridization in polytypic barking frogs. Mol Ecol.

[45] Green RE, Krause J, Briggs AW, Maricic T, Stenzel U, Kircher M (2010). A draft sequence of the Neandertal genome. Science.

[46] Durand EY, Patterson N, Reich D, Slatkin M (2011). Testing for ancient admixture between closely related populatons. Mol Biol Evol.

[47] Freedman AH, Gronau I, Schweizer RM, Ortega-Del Vecchyo D, Han E, Silva PM (2014). Genome sequencing highlights the dynamic early history of dogs. PLoS Genet.

[48] Gompert Z, Lucas LK, Buerkle CA, Forister ML, Fordyce JA (2014). Nice CC Admixture and the organization of genetic diversity in a butterfly species complex revealed through common and rare genetic variants. Mol Ecol.

[49] Eriksson A, Manica A (2012). Effect of ancient population structure on the degree of polymorphism shared between modern human population and ancient hominins. PNAS.

[50] Tuniyev BS, Orlov NL, Ananjeva NB, Agasjan AL (2009). The snakes of the Caucasus.

[51] Nikolsky AM (1916). Reptiles. Faune de la Russie et des pays limitrophes. Vol. 5. Part II.

[52] Nilson G, Tuniyev B, Orlov N, Höggren M, Andrén C (1995). Systematics of the Vipers of the Caucasus: Polymorphism or Sibling Species?. Asiatic Herpetological Research.

[53] Mebert K, Jagar T, Grzelj R, Cafuta V, Luiselli L, Ostanek E, Golay P, Dubey S, Golay J, Ursenbacher S (2015). The dynamics of coexistence: habitat sharing versus segregation patterns among three sympatric montane vipers. Biol J Linn Soc.

[54] Vereshchagin NK (1959). Mammals of Caucasus.

[55] The RW, Genocide C (2013). Genocide, Political Violence, Human Rights Series.

[56] Glotov NV, Severikov LF, Vereshchagin AV (1975). Natural historical and population study of sessile oak (*Quercus petraea* Liebl.) in the North-Western Caucasus. Zh Obshch Biol.

[57] Abbott R, Albach D, Ansell S, Arntzen JW, Baird JE (2013). Hybridization and speciation. J Evol Biol.

[58] ICZN (1999). International Code of Zoological Nomenclature.

[59] IUCN. Standards and Petitions Subcommittee. Guidelines for Using the IUCN Red List Categories and Criteria. Version 11. Prepared by the Standards and Petitions Subcommittee. 2014. http://www.iucnredlist.org/documents/RedListGuidelines.pdf. Accessed 21 Aug 2015.

[60] IUCN. Guidelines for Application of IUCN Red List Criteria at Regional and National Levels: Version 4.0. Gland, Switzerland and Cambridge, UK: IUCN. Available at www.iucnredlist.org/technical-documents/categories-and-criteria. Accessed 21 Aug 2015.

[61] Allendorf FW, Leary RL, Spruell P, Wenburg JK (2001). The problems with hybrids: setting conservation guidelines. Trends in Ecology and Evolution.

[62] Sovic MG, Carstens BC, Gibbs HL (2016). Genetic diversity in migratory bats: Results from RADseq data for three tree bat species at an Ohio windfarm. Peer J.

[63] Sovic M, Fries A, Gibbs HL (2015). AftrRAD: a pipeline for accurate and efficient *de novo* assembly of RADseq data. Mol Ecol Resour.

[64] Rousset F (2008). GENEPOP’007: a complete re-implementation of the GENEPOP software for Windows and Linux. Mol Ecol Resour.

[65] Excoffier L, Lischer HEL (2010). Arlequin suite ver 3.5: A new series of programs to perform population genetics analyses under Linux and Windows. Mol Ecol Resour.

[66] Stamatakis A (2014). RAxML Version 8: a tool for phylogenetic analysis and post-analysis of large phylogenies. Bioinformatics.

[67] Pritchard JK, Stephens M, Donnelly P (2000). Inference of population structure using multilocus genotype data. Genetics.

[68] Jombart T (2008). adegenet: a R package for the multivariate analysis of genetic markers. Bioinformatics.

[69] Jombart T, Devillard S, Balloux F (2010). Discriminant analysis of principal components: a new method for the analysis of genetically structured populations. BMC Genet.

[70] Earl DA, von Holdt BM (2012). STRUCTURE HARVESTER: a website and program for visualizing STRUCTURE output and implementing the Evanno method. Conserv Genet Resour.

[71] Jombart T. A tutorial for Discriminant Analysis of Principal Components (DAPC) using adegenet 2.0.0. 2015. Available at http://adegenet.r-forge.r-project.org/files/tutorial-dapc.pdf. Accessed 21 Aug 2015.

[72] Meyer M, Kircher M, Gansauge MT, Li H, Racimo F, Mallick S (2012). A high-coverage genome sequence from an archaic denisovan individual. Science.

[73] The Heliconius Genome Consortium (2012). Butterfly genome reveals promiscuous exchange of mimicry adaptations among species. Nature.

