# Supplementary material for: Hybrid origin of European Vipers (Vipera magnifica and Vipera orlovi) from the Caucasus determined using genomic scale DNA markers
Source: BMC Evol Biol. 2016 Apr 12;16:76. doi: 10.1186/s12862-016-0647-7 (PMC4828770; doi:10.1186/s12862-016-0647-7)
Supplement: Additional file 1: — D-test and admixture proportion f [46] for V. kaznakovi, V. renardi (including V. lotievi), V. orlovi, and V. magnifica. Each row represents data from all combinations of possible P1, P3, and the Outgroup individuals, where P1 is always represented by V. kaznakovi and P3 is always represented by V. renardi. (DOC 96 kb) [file 12862_2016_647_MOESM1_ESM.doc]

Additional file 1. D-test and admixture proportion *f* (Durand et al., 2011) for *V. kaznakovi,* *V. renardi* (including *V. lotievi*), *V. magnifica,* and *V. orlovi* samples,. Mean±SD, Min-Max. Each row represents data from all combinations of possible P1, P3, and outgroup individuals, where P1 is always represented by *V. kaznakovi* and P3 is always represented by *V. renardi*.

| Group/  Individual | Number of tests, N | Patterson D, averaged across all tests | Number of ABBA sites, averaged across all tests | Number of BABA sites, averaged across all tests | Admixture proportion,  f, % |
| --- | --- | --- | --- | --- | --- |
| Kz2 | 4680 | -0.09±0.38  -1-0.71 | 5.46±3.05  0-15 | 6.19±2.51  1-16 | -0.96±5.68  -15.75-11.81 |
| Kz3 | 4680 | -0.29±0.25  -1-0.6 | 3.70±1.66  0-11 | 6.94±2.84  1-16 | -4.27±3.85  -15.76-5.25 |
| Kz4 | 4680 | 0.06±0.30  -0.83-1 | 6.61±1.92  1-13 | 6.11±2.62  0-15 | 0.66±4.83  -14.44-11.81 |
| Kaz16 | 4680 | 0.02±0.36  -1-1 | 6.26±2.24  0-14 | 6.15±2.59  0-14 | 0.18±6.94  -18.89-17.18 |
| Kaz17 | 4680 | 0.35±0.24  -0.38-1 | 9.35±2.13  4-17 | 4.63  2.07  0-11 | 8.13±  5.27  -8.59-20.61 |
| Kz20 | 4680 | 0.00±0.31  -0.75-1 | 5.59±1.68  1-11 | 5.91±2.54  0-17 | 0.42±4.40  -15.76-10.50 |
| Kz36 | 4680 | -0.05±0.31  -1-1 | 5.17±1.86  0-12 | 5.90±2.43  0-14 | -0.96±4.30  -13.13-10.50 |
| Kz39 | 4680 | -0.01±0.31  -1-0.78 | 5.40±2.02  0-13 | 5.71±2.58  1-14 | -0.41±4.20  -13.13-10.50 |
| All *V. kaznakovi* | 37440 | 0.00000±0.35  -1.0 – 1.0 | 5.94±2.60  0-17 | 5.94±2.60  0-17 | 0.00±5.36  -15.76-15.76 |
| Lo24 | 4992 | 0.85±0.07  0.61-1.00 | 73.25±8.35  56-117 | 6.00±2.66  0-14 | 87.99±13.32  57.57-153.08 |
| Lo71 | 4992 | 0.91+-0.06  0.70-1.00 | 81.50+-8.03  58-111 | 3.80+-2.34  0-12 | 101.65+-12.55 66.73-145.23 |
| Lo72 | 4992 | 0.91+-0.06 0.70-1.00 | 82.00+-8.11  56-109 | 3.84+-2.30  0-13 | 102.26+-12.63 65.42-142.62 |
| Lo74 | 4992 | 0.92+-0.06 0.70-1.00 | 83.76+-8.96 61-113 | 3.48+-2.44  0-13 | 105.05+-14.05  71.96-147.85 |
| Lo75 | 4992 | 0.90+-0.06  0.70-1.00 | 81.65+-8.46  58-111 | 4.39+-2.48  0-13 | 101.09+-13.37 66.73-145.23 |
| Lo76 | 4992 | 0.89±0.05  0.67-1 | 82.5±7.99  60-120 | 4.79±2.21  0-13 | 101.68±12.20  69.34-157.01 |
| Re9 | 4992 | 0.91+-0.05 0.72-1.00 | 78.14+-7.40  57-114 | 3.71+-1.90 0-12 | 97.38+-10.90  69.35-149.16 |
| Re13 | 4992 | 0.93+-0.04  0.76-1.00 | 80.45+-7.71  59-120 | 2.99+-1.79  0-11 | 101.35+-11.12  69.35+-157.01 |
| Re572 | 4992 | 0.92+-0.04  0.73-1.00 | 80.12+-7.77  60-113 | 3.38+-1.77  0-11 | 100.42+-11.13  69.34-147.85 |
| Re62 | 4992 | 0.92+-0.04  0.77-1.00 | 82.93+-7.64  61-117 | 3.47+-1.70  0-10 | 103.96+-11.12  71.96-153.08 |
| Re63 | 4992 | 0.91+-0.05  0.70-1.00 | 79.27+-7.84  58-115 | 3.92+-2.19  0-12 | 98.59+-11.36  66.73-150.47 |
| Re107 | 4992 | 0.92+-0.04  0.75-1.00 | 79.62+-7.22  60-114 | 3.46-1.81  0-10 | 99.65+-10.51  70.65-149.16 |
| Re108 | 4992 | 0.91±0.05  0.73-1.00 | 79.29+-7.05  59-112 | 3.67+-2.00 0-12 | 98.94+-10.31  70.65-146.54 |
| All *V. renardi* and *V. lotievi* | 64896 | 0.91±0.056 0.61-1.0 | 80.34±8.31  56-120 | 3.91±2.26  0-14 | 99.99 |
| mag2 | 4989 | 0.24±0.24  -0.5-0.86 | 9.56±2.31  3-18 | 6.05±2.62  1-16 | 6.03±5.94  -13.74-20.62 |
| mag5 | 4989 | 0.25±0.23  -0.57-0.86 | 9.87±2.84  2-20 | 5.94±2.16  1-14 | 6.75±6.14  -13.74-22.33 |
| orl3 | 4992 | 0.49±0.17  -0.07-0.89 | 17.48±2.42  0-25 | 6.21±2.61  1-16 | 19.35±5.62  -3.44-34.36 |
| orl4 | 4992 | 0.42±0.19  -0.23-0.90 | 13.81±2.90  5-22 | 5.65-2.10  1-14 | 14.03±6.38  -5.15-32.64 |
| orl5 | 4992 | 0.48±0.18  -0.29-0.89 | 16.53±3.73  6-27 | 5.70±2.02  1-13 | 18.62±7.61  -8.59-37.80 |
| orl7 | 4992 | 0.43±0.18  -0.29-0.90 | 13.83±2.39  5-21 | 5.65±2.04  1-12 | 14.06±5.67  -6.87-32.64 |
| orl13 | 4992 | 0.55±0.18  -0.09-1 | 16.54±2.57  8-24 | 4.96±2.30  0-13 | 19.90±6.09  -3.43-36.08 |
| orl15 | 4979 | 0.49±0.21  -0.15-1 | 16.18±3.43  7-29 | 5.50±2.46  0-15 | 18.36±8.18  -6.87-41.23 |
| orl19 | 4992 | 0.53±0.17  0.03-1 | 17.65±2.51  10-25 | 5.62±2.69  0-15 | 20.68±5.63  1.72-34.36 |
| orl20 | 4992 | 0.49±0.16  -0.08-0.92 | 18.36±2.75  10-27 | 6.33±2.39  1-16 | 20.68±6.38  -3.44-41.23 |
| orl29 | 4992 | 0.58±0.15  0.07-0.92 | 20.27±3.03  11-27 | 5.48±2.24  1-14 | 25.40±6.63  3.44-41.23 |
| orl30 | 4992 | 0.56±0.13  0.08-0.90 | 18.10±2.76  7-28 | 5.11±1.72  1-11 | 22.32±5.71  1.72-39.51 |
| orl31 | 4964 | 0.43±0.19  -0.17-0.90 | 13.84±2.64  5-22 | 5.61±2.13  1-14 | 14.14±6.12  -5.15-30.92 |
| orl32 | 4988 | 0.56±0.18  -0.06-1 | 16.96± 2.74  8-26 | 4.96±2.29  0-13 | 20.62±6.45  -1.72-37.79i |
